# clickBrick prompt engineering: optimizing large language model performance in clinical psychiatry

**DOI:** 10.1038/s44184-026-00224-3

**Published:** 2026-06-25

**Authors:** Falk Gerrik Verhees, Fabian Huth, Vincent Meyer, Fabian Wolf, Michael Bauer, Andrea Pfennig, Philipp Ritter, Jakob Nikolas Kather, Isabella Catharina Wiest, Pavol Mikolas

**Affiliations:** 1 Department of Psychiatry and Psychotherapy, University Hospital Dresden, TUD Dresden University of Technology, Dresden, Germany; 2 Department of Medicine II, Medical Faculty Mannheim, Heidelberg University, Mannheim, Germany; 3 National Center for Tumor Diseases, Heidelberg University Hospital, Heidelberg, Germany; 4 Department of Medical Oncology, Heidelberg University Hospital, Heidelberg, Germany; 5 Department of Medicine I, University Hospital Dresden, TUD Dresden University of Technology, Dresden, Germany; 6 Else Kroener Fresenius Center for Digital Health, TUD Dresden University of Technology, Dresden, Germany; 7 Department of Psychiatry and Psychotherapy, Jena University Hospital, Jena, Germany

**Keywords:** Computational biology and bioinformatics, Engineering, Health care, Mathematics and computing

## Abstract

Prompt engineering has the potential to enhance large language models’ (LLM) ability to solve tasks through improved in-context learning. In clinical research, the use of LLMs has shown expert-level performance for a variety of tasks ranging from pathology slide classification to identifying suicidality. We introduce clickBrick, a modular prompt-engineering framework, and rigorously test its effectiveness. Here, we explore the effects of increasingly structuring prompts with the clickBrick framework for a comprehensive psychopathological assessment of 100 index patients from psychiatric electronic health records. We compare the performance of two locally-run LLMs against an expert-labeled ground truth for a variety of successively built-up prompts for the extraction of 12 transdiagnostic psychopathological criteria. Potential clinical value was explored by training linear support vector machines on outputs from the strongest and weakest prompts to predict discharge ICD-10 main diagnoses for a historical sample of 1692 patients. We could reliably extract information across 12 distinct psychopathological classification tasks from unstructured clinical text with balanced accuracies spanning 71% to 94%. Across tasks, we observed a substantially improved extraction accuracy (between +19% and +36%) using clickBrick for the most reactive model. The comparison unveiled great variations between prompts with a reasoning prompt performing best in 7 out of 12 domains. Clinical value and internal validity were approximated by downstream classification of eventual psychiatric diagnoses for 1,692 patients. Here, clickBrick led to an improvement in overall classification accuracy from 71% to 76%. ClickBrick prompt engineering, i.e., iterative, expert-led design and testing, is critical for unlocking LLMs’ clinical potential. The framework offers a reproducible, explainable pathway for deploying trustworthy generative AI across mental health and other clinical fields.

## Introduction

**Fig. 1:**
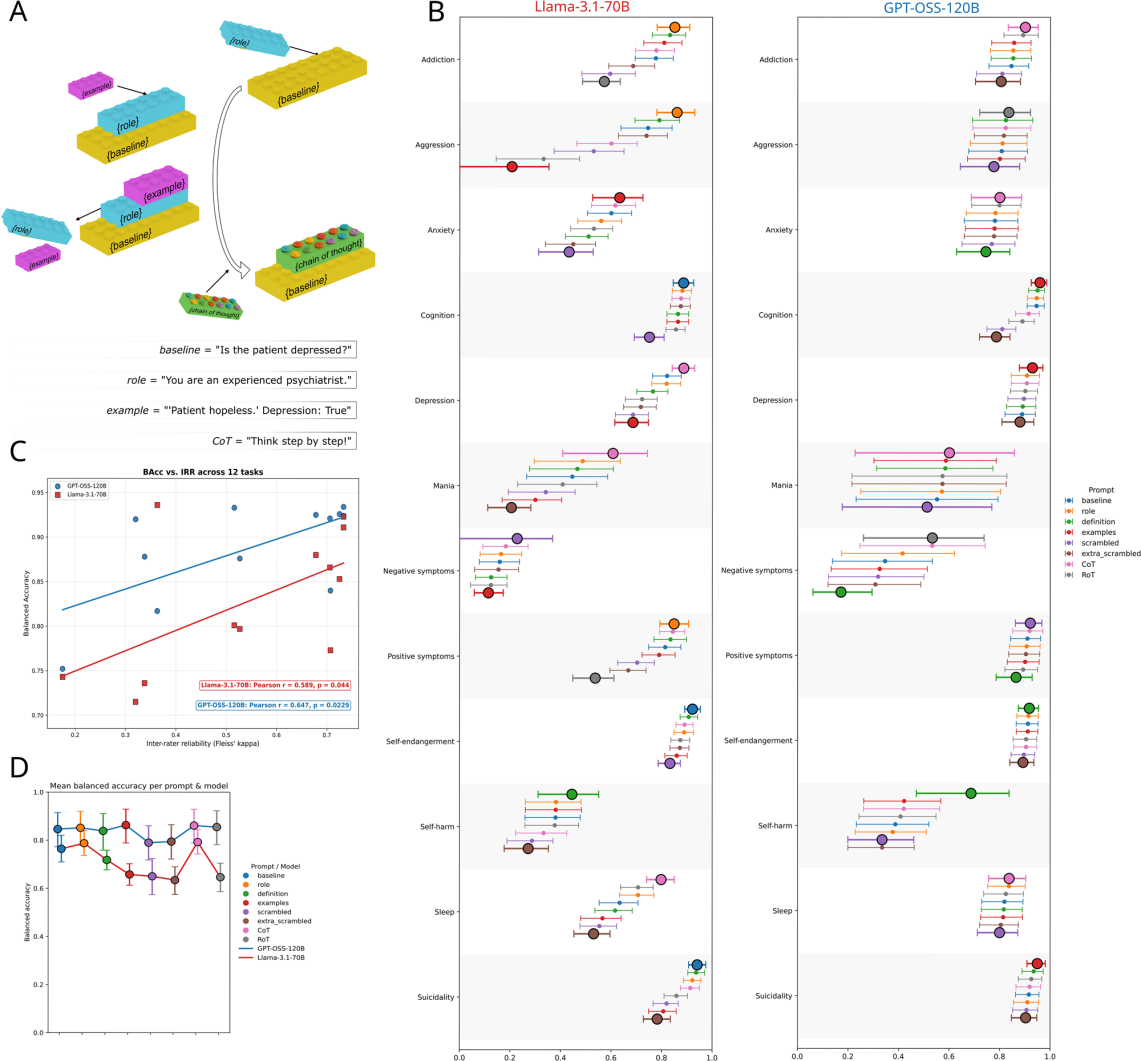
Information extraction from medical free text using the ClickBrick approach. Pipeline overview and performance dteails across psychopathological domains. **A** ClickBrick approach: successively more structured prompts are designed. A task-defining baseline is complemented by role, example and CoT-prompts, as indicated by the colored bricks. Full prompt stack in Supplementary Table S1. **B** Balanced Accuracy (BAcc) for both Llama and GPT models across all domains showing results for all prompts, highlighting best and worst prompting strategies (bold), error bars marking 95%-CIs from 2000-fold bootstrapping across three independent experimental runs. BAcc_Best_ is significantly higher than BAcc_worst_ with *p* < 0.001 for all domains (bar Aggression and Mania with GPT). *P*-values were Benjamini-Hochberg-adjusted across all domains and metrics (false-discovery-rate ≤ 0.05). **C** IRR and BAccs per task are correlated with medium strength in both models (Llama Pearson’s r = 0.589, *p* = 0.044; GPT Pearson’s *r* = 0.647, *p* = 0.023). **D** Ranking of prompt BAccs. CoT: Chain-of-Thought. RoT: Reflection-of-Thought^3^. OLS: ordinary least squares. IRR: Inter-rater-reliability. See Supplementary Table S2 and Supplementary Fig. S2 for other performance metrics.

Generative artificial intelligence (AI) and large language models (LLMs) offer many applications across all domains of medicine from cancer classification in pathology^1^ to identification of suicidality in electronic health records (EHR) in psychiatry^2^. While they are often highly accurate and reliable, it is rarely reported in detail how the design choices that led to the presented results were made - be it by training a foundation model from scratch, fine-tuning an existing model on relevant data, or by engineering prompts for in-context learning. Briefly, prompt engineering (PE) involves describing a task in natural language to guide an LLM in completing it^3^. In principle, it is an efficient solution^4^ that should be thoroughly evaluated before opting for another strategy. The relevance of structured PE is further underscored by the rapid depreciation of static rules on prompt engineering with new model releases^5^. Recently updated guidance on prompting by Google and Anthropic^6,7^, two makers of such models, underscore this dynamic development. Despite growing LLM use, there is no consensus on how to evaluate or structure prompts in medical applications^2–4,8,9^. Given these challenges, a modular and testable approach is needed. To address this dilemma, we introduced clickBrick, a systematic, reproducible framework for PE in medicine, which enables direct comparison of prompt formats, including few-shot and chain-of-thought prompts^3^, illustrated across 12 clinical information extraction tasks from unstructured medical free text. Those tasks cover the whole of psychopathology, ranging from substance use to suicidality in a transdiagnostic, holistic representation of patient behavior, cognition and emotion^10^. clickBrick proposes a Lego-style, step-wise protocol for designing prompts that guide large language models (LLMs) in clinical work (Fig. 1A). This manual approach directly incorporates expert knowledge of real-world problems, increases transparency and alleviates the need for post-hoc explanations. For a description of all relevant prompts across all 12 tasks see Supplementary Table S1. Finally, the designer benchmarks every prompt configuration against an expert-curated ground truth, ideally verified by at least 3 blinded subject matter experts with consensus, majority, or combined method resolution^11^ - to discover the most reliable prompt stack. To scale analysis of unstructured medical text without the need for complete subject-matter expert annotation, we suggest a prediction of available structured information, like ICD-10 diagnosis to compare most vs. least accurate prompting strategies as a demonstration of the usefulness of such systematic prompt engineering.

## Methods

### Systematic prompt evaluation and optimization

Expert-crafted prompts are decomposed into their semantic components and their combinations subsequently tested and compared as prompt (Fig. 1A), programmatically combined (see code repository) and automatically sent to the LLM. The researcher begins with a “zero-shot” *baseline task query* (“Does the patient show symptoms of depression?”) and then *clicks on successive prompt-bricks* that add (a) a *professional role* (“You are an experienced psychiatrist.”), (b) “one-shot” concise *operational definitions* of the diagnostic target (“Depression is characterized by sad mood, reduced energy or drive, and a loss of interest.”), (c) “few-shot” *labeled examples* of e.g., positive and negative cases (“‘The patient displays elevated mood.’ → no depressive symptom, ‘The patient felt increasingly sad and hopeless.’ → depressive symptoms present”), (d) and an explicit request for *Chain-of-Thought (CoT) reasoning* (“Think step by step!”). The clickBrick approach was also extended to a Chain-of-Thought and a simple Reflection-of-Thought prompt^3^. For a full list of all prompts see Supplementary Table S1. All prompts were tested between two open-weight LLMs (Llama-3.1-70B-Instruct^12^ and GPT-OSS-120B)^13^ locally run with the llama.cpp^14^ project and compared for their accuracy, balanced accuracy (BAcc), precision, recall and F1 score, with BAcc as preferred measure for imbalanced categories. The authors assert that all procedures contributing to this work comply with the ethical standards of the relevant national and institutional committees on human experimentation and with the Helsinki Declaration of 1975, as revised in 2013. All procedures involving human subjects/patients and their data were approved by the ethics committee of Technical University Dresden, reference number BO-EK-400092023. Informed consent was not required for this study because the research involved data from which all personal identifiers had previously been removed. The design of the study followed TRIPOD-LLM guidelines^15^ and ensured that there was no interaction or intervention with participants and no potential for harm or invasion of privacy.

### Definition of transdiagnostic information extraction tasks

A transdiagnostic approach to psychopathology aims to describe and combine traits and states to enable better description of patient presentation with potential to identify subgroups and treat them with greater precision^16^. Here, we use an adapted paradigm^10^ evaluating 12 dimensions of psychopathology: anxiety, addiction, cognition, depression, hostility, mania, negative and positive symptoms, sleep and self harm (with the dimensions self-endangerment, non-suicidal self harm, and suicidality).

### Ground truth curation

For a dataset of 2520 cases from 1692 individual patients, admission notes from the electronic health records of a large German supra-maximum psychiatric acute care ward (all patients from 01 Jan 2022 to 31 May 2024) were analyzed with a locally run and privacy preserving pipeline^17^ to extract a binary evaluation (present/not present) on each of the transdiagnostic parameter from the initial medical evaluation (with the absence of reporting on the parameter being interpreted as not present). Prompts were optimized for the highest identification balanced accuracy compared against a thrice independently rated ground truth for a sub-sample of 100 cases (01 Jan to 31 Dec 2023) by 3 subject-matter experts (FGV: resident psychiatrist, VM: trained research assistant, PM: attending psychiatrist), for detailed sample characteristics see Wiest and colleagues^2^. Consensus was reached by majority vote and, in situations of parity, by structured discussion. For each of the 12 symptom domains, we assessed inter-rater reliability across the three independent clinicians in two complementary ways. First, we quantified absolute (raw) agreement as the proportion of subjects whose three ratings were identical (false, true, not described in source data). Second, chance-corrected reliability was estimated with Fleiss’ κ, appropriate for 3 raters and nominal data.

### Statistical analysis

If not indicated otherwise, uncertainty was quantified via non-parametric bootstrapping test: 2000 subject-level resamples with replacements were drawn, κ and BAcc, respectively, recomputed for each, and the 2.5^th^ and 97.5^th^ percentiles of the bootstrap distribution reported as the 95% confidence interval. We assessed the association between rater agreement and task accuracy by computing Pearson product–moment correlation coefficients for Fleiss’ κ with BAcc for all 12 domains. Additionally, the most accurate prompting strategy is compared to the least accurate one for the ensuing classification task (see below) to gauge the relevance of information extraction accuracy, with a two-sided Wilcoxon signed-rank to determine significant differences across folds. Throughout, multiple testing was addressed with Benjamini–Hochberg adjustment for a false-discovery-rate ≤ 0.05. All calculations and performance evaluations were done using pandas 2.2.3^18^, numpy 2.2.0^19^, statsmodels 0.14.4^20^, and Scikit-learn 1.6.0^21^ packages in Python 3.12.3^22^. The analytic code is available at: https://github.com/verrik/clickBrick_PromptEngineering. The pipeline for local deployment of LLMs in a privacy-preserving clinical setting was described elsewhere^23,24^ and is available here: https://github.com/KatherLab/LLMAIx. Inferences with the local models were run on one Nvidia A6000 GPU (48BG VRAM) on hospital premises (with max. token length of 2048, chosen to accommodate note length). Hyperparameter were not tuned but set for deterministic results (temperature 0.0, repeat penalty 1). For an exemplary, fictional case vignette see Supplementary Table S1.

### Prediction of diagnosis at discharge using machine learning

We employed a linear support vector machine to predict eventual ICD-10 psychiatric primary diagnoses (implemented in NeuroMiner 1.2^25^, MatLab 9.14.0.2206163 (R2023a)^26^, grouped by the 6 main diagnostic groups represented in our sample F0-F4 (F0 - dementias, F1 - substance use disorders, F2 - psychotic disorders, F3 - affective disorders, F4 - anxiety disorders, and F6 (personality disorders, here exclusively borderline PD), excluding the groups not represented in our study sample below a threshold of 50 cases (detailed description see Supplementary Fig. S1). The model was trained on 2520 cases from 1692 individual cases using 12 binary features (0/1) reflecting key psychopathological domains (e.g., suicidality, addiction, depression), derived from clinician letters via LLM-based annotation. Classification followed a one-vs-rest strategy for six diagnostic categories (F0, F1, F2, F3, F4, F6), and for each, two models were run: one using features from the best-performing LLM prompt (based on balanced accuracy), and one using the worst. To mitigate class imbalance, ranging from 5.60% (F6), 5.79% (F4), 8.37% (F0), 22.7% (F2), 25.6% (F3), to 30.44% (F1), we applied instance-weighted hyperplane adjustment via the LIBSVM 3.1.2 implementation (C-SVC with L1-loss), ensuring minority classes received proportionally higher weighting during optimization. We did not apply oversampling or undersampling for class balancing, as such techniques have been questioned for potentially reducing model generalizability and performance^27^. We used nested 10-fold cross-validation to prevent information leakage, enable hyperparameter tuning, and ensure reliable generalization. No imputation was necessary due to complete data. The SVM regularization parameter (C) was tuned across 11 logarithmically spaced values ranging from 0.015625 to 16. No filter or wrapper-based feature selection was performed given the low-dimensional feature space relative to the sample size. The case-to-feature ratio was 210:1 (2520 cases and 12 features), supporting stable model estimation without risk of overfitting.

## Results

### Transdiagnostic information extraction is influenced stronger by prompt engineering for a smaller model

Overall, clickBrick increased the mean BAcc by 26.5% from 56.2% (95%-CI 53.3–59.1%) with the worst prompts to 82.8% (95%-CI 81.2–84.4%) with best for the Llama model, and a more measured increase in BAcc of 12.0% for the larger GPT model. The largest increases were found in the Llama model, specifically for depressive symptoms (36%), mania (32%) and cognitive impairment (30%). Increase above the baseline prompt was on average a more modest, but still meaningful 6.3% from 76.4% (95%-CI 75.9–77.0%) to 82.8% (95%-CI 81.2–84.4%), with largest increases for impaired sleep (23.4%), depressive symptoms (10.2%) and aggression (6.9%) (Fig. 1B). In accordance with the more recent advent of reasoning models, a simple CoT and a more complex RoT prompt were included in the clickBrick hierarchy. As hypothesized, CoT prompting yielded the highest accuracy in 7 out of 12 domains, and was statistically indistinguishable from the top-performing prompt in 3 additional domains after adjustment (*p* ≥ 0.05, Supplementary Table S2). However, its effectiveness varied strongly by task: CoT was significantly outperformed by a 3-shot prompt for both aggression and self-harm (*p* < 0.05, Supplementary Table S2), underscoring the importance of domain-specific evaluation. What is more, the second reasoning prompt (RoT) that did display superior performance elsewhere^3^ showed poor accuracy in our sample, performing worst for 3 out of 12 domains. Semantic noise in the form of deliberately introduced, script-generated typing errors making the expert-designed prompts harder to interpret by the LLMs^28^ was injected in an attempt to force underperformance for the sake of this argument. Noisy prompts did perform worst in 5 out of 12 domains (1D). For comparison, the GPT model that already incorporates reasoning in its training^13^ showed less sensitivity to prompt engineering, with the best prompt outperforming the baseline only for depression. Inter-rater reliabilities (IRR) ranged from Fleiss’ κ of 0.18 for self-endangerment to 0.73 for aggression and suicidality, designating slight to substantial agreement^29^ (Supplementary Table S2). The best results for BAccs after clickBrick PE were correlated with medium strength to IRR in both models (Llama Pearson’s *r* = 0.589, *p* = 0.044, GPT Pearson’s *r* = 0.647, *p* = 0.023, 1 C).

### Increased discharge diagnosis prediction validates prompt engineering effect beyond the ground truth

**Fig. 2:**
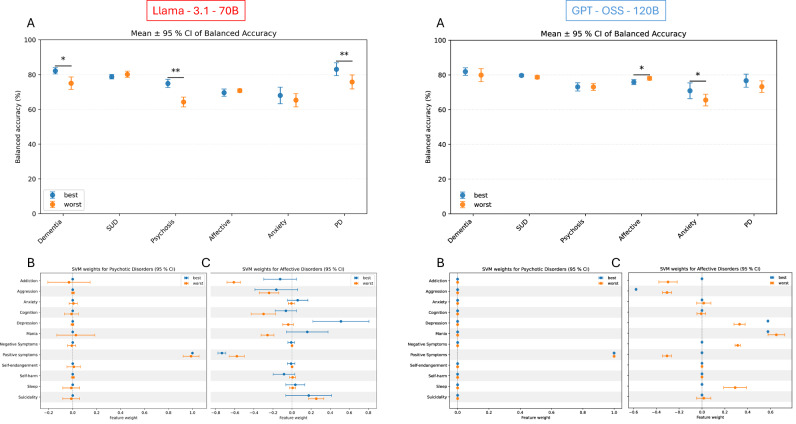
Validation through prediction of diagnosis at discharge using machine learning. Comparison of diagnosis prediction accuracy between tested models, and feature weights for selected diagnostic groups. **A** BAcc achieved by SVM when trained on the least-noisy (“best”) versus the most-noisy (“worst”) version of the LLM-extracted data for each diagnosis group per upstream model. “Best” (blue) and “worst” (orange) mark the mean BAcc across the 10 cross-validation folds with 95%-CI. Black horizontal bars connect paired conditions that differ significantly (two-sided Wilcoxon signed-rank test, *n* = 10, Benjamini-Hochberg adjusted for multiple comparisons). Significance levels indicated by asterisks above the bar (*p < 0.05, **p < 0.01). Selected, instructive feature weights (see Supplementary Fig. S3 for complete weights): **B** SVM feature weights for F2/Psychotic Disorders: “best” and “worst” models both rely mainly on positive symptoms (i.e., hallucinations, imperative voices, etc) for classification. **C** SVM feature weights for F3/Affective Disorders: the “best” model is most influenced by the presence of depression, mania, and suicidality and the absence of positive symptoms in its classification, while the “worst” models rely equally on the absence of positive symptoms, and the presence of suicidality and is influenced more by the absence of mania, cognitive impairment or signs of addiction. For the GPT-based information extraction, “best” and “worst” features converge. See Supplementary Table S3 for full information.

To evaluate potential downstream utility and provide internal validation, we extended our clickBrick-optimized extraction to a dataset of 2520 individual cases from 1692 patients with a mean age of 45.2 ± 0.76 years and a balanced gender distribution (female: 1303 cases, male: 1215 cases, non-binary: 2 cases). Here, we used linear support vector machines (SVM) to predict final diagnosis at discharge from the clinical notes taken at admission to evaluate the optimization’s impact on potential clinical utility. We predicted class-vs-other for all relevant ICD-10 diagnostic groups that were represented in our sample (Supplementary Fig. S1: F1 - 767 cases with substance use disorders, F3 - 645 cases with affective disorders, F2 - 572 cases with psychotic disorders, F0 - 211 cases with dementia, F4 - 146 cases with anxiety disorders, F6 - 141 cases with personality disorders - here exclusively borderline, and 38 other cases). Overall classification performance (in BAcc) increased moderately with the “best” strategy with Llama, rising from 71.9% to 76.2%, and increased little with GPT from 74.7 to 76.4%. Significant improvements in classification were observed for substance use, psychotic and personality disorders, but not for the rest (*p* < 0.05, Benjamini–Hochberg adjusted, (Fig. 2A) with the Llama model, and only for anxiety disorders with GPT. We subsequently analyzed feature weights to identify the clinical domains the SVM relied on most: the larger (in absolute value) the weight, the more that domain drives the decision. For psychotic disorders (Fig. 2B), positive symptoms carry the top weight in both the “best prompt” and “worst prompt” models, confirming their central diagnostic importance. Yet model quality is greatly improved from a BAcc of 64% for “worst” to 75% for “best” prompting strategy with Llama (and no significant change with GPT), showing that upstream clickBrick, not a different leading feature, explained the performance gain. Conversely, for affective disorders the distribution of feature weights shifts substantially between models (Fig. 2C). Despite this shift, classification performance remained statistically indistinguishable between the “worst” and “best” prompting strategies.

## Discussion

We show here that LLMs in clinical psychiatry offer a way to rapidly and safely aggregate and evaluate available information, e.g., from EHRs. Conversely, failure to synthesize relevant information delays diagnosis and thus treatment and often leads to considerable suffering, especially for patients with mental disorders like bipolar disorder or schizophrenia, which take between approximately 1 and 6 years to correct diagnosis^30,31^. With clickBrick, we provide a way to optimize LLMs for the challenge of predictive modeling based on EHR to better characterize the “deep patient”^32^. The unstructured free text thus made available encapsulates highly ecologically valid clinical data^2,23^. We offer a starting point for future systematic evaluation of LLMs for information extraction, and in principle for any task that is defined by natural language or in fact multi-modal prompts, e.g., for pathological classification^1^. ClickBrick addresses the currently highly heterogeneous reporting quality of prompting strategies^2,3^. It encourages systematic and transparent iterative prompt engineering to enable better model performances. Recent evidence of sensitivity to prompt engineering even in new and large models^33^ underlines the relevance of a framework like clickBrick and supports our finding improved performance in both tested models, albeit to varying degrees. Our proposed approach to PE can thus be considered a relevant means of improving the systematic evaluation of in-context learning: while we were consistently able to identify prompts for improved information extraction accuracy across all domains of interest, we could not deduct general heuristics for optimal prompts across all tasks. A large benefit derived indeed from identifying *under-performing* prompts that were *prima-facie expected to work better*, like few-shot prompting with added examples or the RoT reasoning prompt. And although the CoT reasoning prompt performed mostly superior (e.g., for anxiety), simpler prompts did better for several other psychopathological constructs, including the baseline prompt for suicidality. At the same time, we could not reach a consistently high accuracy across all 12 domains, ranging from 71% (negative symptoms) to 94% (mania). Effects of PE were less pronounced though still clearly distinguishable for the larger reasoning model GPT-OSS-120B. The medium-strength positive correlation between inter-rater reliability and extraction accuracy across tested models offers a potential explanation for both observations: when high rater agreement denotes low classification difficulty for subject-matter experts, a higher LLM extraction accuracy can be expected, and vice versa. This assumption might be tested in future research. In direct comparison, the increase of the PE effects inversely with model size suggests, that orchestrated agentic architectures, which rely on smaller models for tasks within clinical or research workflows will profit greatly from clickBrick-style PE at the handover to smaller models, as might already be the case with “skills” in recent agentic systems^34^. Smaller language models can be more suitable and are usually more economical than overall more capable general-purpose LLMs for specific or repetitive tasks, with heterogeneous systems using larger models only when needed^35,36^.

Downstream classification of diagnostic groups based on “best” and “worst” extraction strategies was improved by clickBrick optimization for the smaller model, though not for all psychopathological domains, providing some internal validation on a larger dataset of patient cases. The observation of shifting feature weights suggests that the classifier may have relied on different information in response to reduced extraction fidelity. For the larger GPT model, “best” and “worst” features converge, in line with the smaller influence of PE in information extraction accuracy described above.

In summary, we do show that comprehensive trans-diagnostic patient psychopathologies can be extracted from EHRs with good to excellent performance, which extends the existing knowledge on the capabilities of LLMs in psychiatry beyond single symptoms or social determinants of health^2,37^. Compared to traditional natural language processing methods^38^, there appear to be no principal limits to LLM applicability while their inclusivity using open-source resources and limited need for computer sciences experience makes them in principle applicable throughout different health care settings, and languages, as successful deployment in this mono-centric context suggests.

Note that clickBrick does not provide a definitive method of identifying the globally best prompt for any given task, only the best one in the local distribution of expert-designed prompts. The merit of this approach appears to be a high consistency across tasks^39^, which is also evident in this work with high accuracies larger than 71%. Still, an alternative approach would be the automated generation and subsequent testing of prompt variations by other LLMs^40–42^, e.g., based on only the initial baseline prompt, as has been described for studies with no medical background^40^, on partly synthetic clinical data^43^, or static medical benchmarks^44,45^. While this will introduce other limitations (like overfitting to the ground truth sub-sample), we encourage further research into such an automated approach to clickBrick prompting, when coupled with rigorous external validation. We maintain nonetheless that manual, expert-led prompt design excels in providing reproducibility and explainability based in understanding of the use case at hand, without the need to validate prompt constructs afterwards. Finally, while we did address a core ethical consideration by safeguarding data privacy with locally hosted models on hospital premises, we could not systematically assess model biases. And while we identified patient preferences for academic-led exploration of AI driven data analysis over industry research in mental health^46^, we did not involve experts with lived experience in the development of clickBrick. We thus recommend the analysis of differential performance for subgroups^47^ based for example on gender or ethnicity for any future study that is primarily concerned with real-world applications of LLMs. We further recommend meaningful patient involvement to identify actual unmet needs that AI information extraction from EHRs might help solve, like risk stratification for the prevention of coercive measures. In light of recent alleged harms in association with LLM chatbot and AI character use, this will be increasingly useful to safeguard not only these public-facing, but also more clinical applications of LLMs^48,49^.

In conclusion, this study demonstrates that a systematic prompt engineering framework like clickBrick significantly improves information extraction from clinical free text with LLMs. Thus, for future research incorporating foundational generative AI models, we encourage the inclusion of a transparent reporting strategy on prompt composition, as clickBrick aims to be. Sticking to the same prompt for all domains would have led to performance reductions in information extraction in this study. Consequently, the clickBrick approach to prompting and subsequent reporting taken here might serve as a starting point for future use of natural-language-guided AI in medicine, for the sake of both the uncovering of the most efficient uses of genAI in medicine and of scientific reproducibility in this context.

## Supplementary information

Supplementary information

## Data Availability

The source data are not publicly available because it contains real-world clinical information that could compromise the privacy of research participants.

## Code availability

The information extraction pipeline is publicly available at https://github.com/KatherLab/LLMAIx. The analytic code is available at https://github.com/verrik/clickBrick_PromptEngineering.
