# Supplementary material for: clickBrick prompt engineering: optimizing large language model performance in clinical psychiatry
Source: Npj Ment Health Res. 2026 Jun 25;5:30. doi: 10.1038/s44184-026-00224-3 (PMC13304116; doi:10.1038/s44184-026-00224-3)
Supplement: Supplementary file 1 — Supplementary information [file 44184_2026_224_MOESM1_ESM.pdf]

Supplementary Table S1: Prompts for all domains

**Prompt**	**Addiction**	**Aggression**	**Anxiety**	**Cognition**	**Depression**	**Mania**	**Negative symptoms**	**Positive symptoms**	**Self-endangerment**	**Self-harm**	**Sleep**	**Suicidality**
**baseline (Does the patient …)**	use drugs or alcohol?	show aggressive behaviour towards others?	show symptoms of anxiety?	show cognitive impairment?	show depressive symptoms?	show manic symptoms?	show negative symptoms?	show positive symptoms?	engage in behaviour that endangers themselves?	exhibit self-harming behaviour?	have sleep problems?	exhibit suicidal behaviour?
**role**	You are an experienced, attentive physician with specialized knowledge in psychiatry.											
**definition**	Any recent substance use (alcohol, cannabis, meth, cocaine, heroin, ketamine, etc.). Nicotine and caffeine not counted.	Hostile, irritable, explosive, insulting, or deliberate harm towards others.	Symptoms include somatic symptoms (palpitations, chest pain, shortness of breath, dizziness, muscle tension), nervousness, free-floating anxiety, depersonalization, derealization, fear of dying, fear of losing control, phobias.	Reduced comprehension, concentration, memory deficits.	Defined by ICD-10: low mood, lack of affect, reduced emotional responsiveness, reduced motivation/activity, inability to feel pleasure, loss of interest, fatigue, poor concentration, low self-esteem, guilt, feelings of worthlessness, agitation, loss of libido.	Defined by ICD-10: disproportionate elevated mood, increased drive, heightened sense of vitality, sociability, libido, overconfidence, reduced need for sleep, recklessness, irritability, reduced concentration, logorrhea, delusions of grandeur, hallucinations.	Loss of cognitive/psychomotor abilities: anhedonia, apathy, blunted affect, attention problems, social withdrawal, alogia, psychomotor retardation.	Defined as: ego disturbances (thought insertion, withdrawal, broadcasting), perceptual disturbances/hallucinations (auditory, visual, olfactory, tactile), delusions, formal thought disorder (loosening of associations, blocking, neologisms), bizarre behavior.	Behavior (conscious or unconscious) placing oneself at risk of harm.	Intentional, often repetitive damage to one's own body without suicidal intent. Superficial cuts/scars are typical.	Sleep onset problems, maintenance problems, early waking, poor sleep quality, daytime fatigue, excessive sleepiness. If well-controlled on medication, considered not present.	Thoughts of suicide, planning suicide, recent suicide attempts, or omission of life-saving actions with intent to die.
**examples**	Example 1: Occasional cannabis and meth use → Yes. Example 2: Denies alcohol and drug use → No. Example 3: Admitted intoxicated → Yes.	Example 1: Aggressive and hard to limit → Yes. Example 2: Cannot rule out aggression → Yes. Example 3: No signs of aggression → No.	Example 1: No anxiety or compulsions → No. Example 2: Fear of failure and disappointing family → Yes. Example 3: Suspicious, anxious, nervous → Yes.	Example 1: Slightly reduced concentration → Yes. Example 2: Fully oriented, no deficits → No. Example 3: Slightly impaired after alcohol intoxication → Yes.	Example 1: Patient is withdrawn, unresponsive → Yes. Example 2: Friendly and outgoing → No. Example 3: Mood appropriate → No.	Example 1: Patient is irritable, elevated mood → Yes. Example 2: Increased drive, confrontational → Yes. Example 3: Patient friendly and calm → No.	Example 1: Hardly leaves home, reduced family contact → Yes. Example 2: Psychomotor retardation, apathy → Yes. Example 3: Open and responsive → No.	Example 1: Patient reports hearing voices and delusions → Yes. Example 2: Acute intoxication with psychotic experience → Yes. Example 3: No hallucinations or delusions → No.	Example 1: Acutely endangered due to misperception → Yes. Example 2: No signs of self-endangerment → No. Example 3: Found disoriented in traffic → Yes.	Example 1: Self-harm scars present → Yes. Example 2: Skin intact → No. Example 3: Recently self-harmed due to stress → Yes.	Example 1: Poor sleep, frequent waking → Yes. Example 2: No sleep problems → No. Example 3: Controlled with medication → No.	Example 1: Tried to jump from bridge → Yes. Example 2: Expresses wish to die → Yes. Example 3: No suicidal thoughts → No.
**scrambled**	DEOS ThE	dOEs ThE	does THE	dEos tHE	dOES The	deOS THE	dOes THe	DOES ThE	dOEs The	dOEs tHE	does THE	dEos THe

	PATIEnt uSE drgUs OR AcOlhLO?	pIATENT SOhW aGgRESSiVe bAEoVHIR TaWDRos otHERs?	PaTIENt SHOW SyMpTOMs oF ANXieTY?	PIETNaT shOW CIGtOvNIE InmTEIRaPM?	PnIAett sHOw DISPrvESEE sYmptOMS?	PaTIenT sHOW MANIC SyMPTOMS?	PATIENt SoHw neGaTiVE SYMPToMs?	PNiATEt ShoW PtIvSOiE sMMtYPOS?	PaTEINT EnGAGE iN BAiOhvEr THaT endANgERS TelmESSEVH?	pENAiTt EbXHIIT SHNAErI-FmLG bveaRoih	PnEitAT HAvE SLEEP PRoBlEmS?	paTnEIT eBiIxHT siuIaCdL bERVHaIO?
**extra scrambled (i.e., +role)**	yoU ARE AN eDEIEXNEPCR, ATTeNTiVE PyhsSCCin WiTh SALEiEZCiPD kLeGWnDOE in PSyChIATry.											
**CoT**	Think step by step!											
**RoT**	Simulate the thought process of three independent psychiatric experts analyzing the given report. Each expert should follow a step-by-step reasoning process from Step 1 to Step X and answer the question independently. After completing their individual analyses, the experts should engage in a discussion, review each other's thought processes, and collaboratively reach a final consensus. Clearly present the individual thought process of each expert, followed by the group discussion and the final conclusion.											
**case vignette**	Patient phoned the regional crisis line after escalating suicidal ideation with a specific plan (overdose on prescribed venlafaxine). Crisis clinician coordinated EMS transport. On scene, patient was tearful, expressed hopelessness, but denied attempts that day. Collateral from friend corroborates 2-week functional decline, social withdrawal, and missed workdays. Prior Psychiatric Treatment & Course: Out-patient: Diagnosed with Major Depressive Disorder (recurrent, moderate) at age 25; managed by GP with sertraline 50 mg daily (poor adherence in past 3 months). Psychotherapy: Six CBT sessions (2019) – self-terminated due to scheduling conflicts. Hospitalizations: None. Substance Use: Social alcohol (1–2 drinks/week); denies illicit substances or tobacco. Medical History: Hypothyroidism (on levothyroxine 75 µg daily, TSH last month 2.1 mIU/L); no surgeries. Family & Social History Family Psychiatric Hx: Mother with recurrent MDD; paternal uncle died by suicide (age 48). No bipolar or psychotic disorders reported. Developmental / Education: Unremarkable childhood, university degree in graphic design. Occupational: Full-time freelance designer; workload recently decreased. Living Situation: Rents one-bedroom apartment; single, limited social support. Protective Factors: Strong attachment to pet dog, stated desire to “not hurt my mother,” insight into illness, willingness to engage in treatment. Mental Status & Psychopathology Assessment. Appearance: Thin, casually dressed, fair hygiene, slumped posture. Behavior: Psychomotor retardation; cooperative, maintains eye contact. Speech: Soft, slow, decreased spontaneity. Mood: “Empty, exhausted”. Affect: Constricted, congruent with mood. Thought Process: Linear, goal-directed. Thought Content: Worthlessness, excessive guilt, passive SI progressing to active SI with plan; no homicidal ideation, delusions, or obsessions. Perception: No hallucinations. Cognition: Alert & oriented ×4; attention intact; immediate recall 3/3, 3/3 after 10 minutes. Insight/Judgment: Good insight into depressive symptoms; judgment impaired by hopelessness around future. Physical Examination (Admission): Vital Signs: BP 118/72 mmHg, HR 76 bpm, Temp 36.7 °C, RR 14 /min, SpO_2 98 % RA. General: Appears stated age, no acute distress. Pupils equal, round, reactive; TMs intact; oropharynx moist. Cardiovascular/Respiratory: normal sounds(S1/2), no murmurs; lungs clear bilaterally. Abdomen: Soft, non-tender, normoactive bowel sounds. Neuro: Cranial nerves I–XII intact; motor 5/5; reflexes 2+ symmetric; gait normal. Skin: No rashes, track marks, or self-harm scars.											

A string would be handed to the large language model, comprising the case report and the prompt (“{report} {prompt}”) as one continuous text. All prompts were expert-designed to give the model relevant knowledge that a human rater would benefit from. Definitions are based on ICD-10[1] and AMDP[2] manuals. Intentional noise was introduced for the two scrambled prompts in the expectation of reduced performance, based on a script to experimentally circumvent proprietary model guardrails[3]. Chain-of-Thought (CoT) and Reflection-of-Thought (RoT) prompt structures were adapted from Wang and colleagues[4]. A typical, though fictional case vignette is also shown here.

---

[1] World Health Organization, *ICD-10 : international statistical classification of diseases and related health problems : tenth revision* (World Health Organization, 2004), https://iris.who.int/handle/10665/42980.

[2] Arbeitsgemeinschaft für Methodik und Dokumentation in der Psychiatrie (AMDP), *Das AMDP-System: Manual Zur Dokumentation Des Psychischen Befundes in Psychiatrie, Psychotherapie Und Psychosomatik*, 11. vollständig überarbeitete Auflage (Göttingen: Hogrefe Verlag, 2023), https://www.hogrefe.com/de/shop/default-name-96607.html.

[3] John Hughes et al., “Best-of-N Jailbreaking” (arXiv, December 19, 2024), https://doi.org/10.48550/arXiv.2412.03556.

[4] Li Wang et al., “Prompt Engineering in Consistency and Reliability with the Evidence-Based Guideline for LLMs,” *Npj Digital Medicine* 7, no. 1 (February 20, 2024): 1–9, https://doi.org/10.1038/s41746-024-01029-4.

Supplementary Table S2: Performance metrics and significance levels for all domain extractions

Llama-3.1-70B														
**Condition [total No./100]**	**Prompt**	**Accuracy**	**95% CI**	**BAcc**	**95% CI**	**Precision**	**95% CI**	**Recall**	**95% CI**	**F1**	**95% CI**	**significant ΔBAcc vs. best prompt**	**p-value**	**IRR Fleiss' κ (95% CI)**
**Addiction [38]**	1	0.82	0.04	0.82	0.04	0.73	0.07	0.83	0.08	0.78	0.04	yes	0.018	0.678 (0.577-0.774)
	2	0.89	0.04	0.88	0.04	0.85	0.07	0.85	0.04	0.85	0.05			
	3	0.87	0.04	0.87	0.03	0.80	0.07	0.87	0.00	0.83	0.04			
	4	0.86	0.03	0.84	0.02	0.86	0.07	0.76	0.00	0.81	0.03			
	5	0.75	0.00	0.70	0.01	0.78	0.03	0.48	0.04	0.59	0.02			
	6	0.79	0.04	0.75	0.02	0.78	0.15	0.61	0.08	0.69	0.02			
	7	0.82	0.04	0.82	0.06	0.73	0.01	0.83	0.15	0.78	0.07	no	0.106	
	8	0.69	0.19	0.66	0.21	0.59	0.27	0.55	0.28	0.57	0.27	yes	<0.001	
**Aggression [25]**	1	0.86	0.01	0.84	0.01	0.70	0.04	0.80	0.00	0.75	0.02	no	0.073	0.733 (0.603-0.844)
	2	0.93	0.02	0.91	0.07	0.86	0.03	0.87	0.15	0.86	0.06			
	3	0.87	0.01	0.91	0.01	0.65	0.02	1.00	0.00	0.79	0.02			
	4	0.77	0.00	0.55	0.00	0.75	0.00	0.12	0.00	0.21	0.00	yes	<0.001	
	5	0.79	0.01	0.68	0.03	0.61	0.04	0.47	0.06	0.53	0.04			
	6	0.86	0.03	0.84	0.05	0.68	0.05	0.81	0.11	0.74	0.06			
	7	0.84	0.02	0.72	0.04	0.80	0.12	0.48	0.10	0.60	0.08	yes	<0.001	
	8	0.80	0.02	0.60	0.05	1.00	0.00	0.20	0.10	0.33	0.14			
**Anxiety [31]**	1	0.59	0.01	0.70	0.01	0.43	0.01	1.00	0.00	0.60	0.01	no	0.517	0.338 (0.239-0.438)
	2	0.51	0.01	0.65	0.01	0.39	0.01	1.00	0.00	0.56	0.01			
	3	0.40	0.05	0.57	0.04	0.34	0.02	1.00	0.00	0.51	0.02			
	4	0.73	0.04	0.74	0.03	0.55	0.05	0.74	0.00	0.63	0.03			
	5	0.57	0.06	0.56	0.07	0.37	0.07	0.53	0.12	0.43	0.09			
	6	0.51	0.05	0.55	0.06	0.34	0.04	0.65	0.08	0.45	0.06	yes	<0.001	
	7	0.66	0.05	0.72	0.03	0.48	0.04	0.87	0.08	0.62	0.03	no	0.806	
	8	0.49	0.08	0.61	0.03	0.37	0.03	0.94	0.08	0.53	0.02			

**Cognition [76]**	1	0.83	0.00	0.79	0.00	0.90	0.00	0.87	0.00	0.89	0.00	no	0.796	0.516 (0.352-0.656)
	2	0.82	0.06	0.78	0.05	0.90	0.03	0.86	0.08	0.88	0.04			
	3	0.76	0.00	0.50	0.00	0.76	0.00	1.00	0.00	0.86	0.00	yes	<0.001	
	4	0.76	0.00	0.50	0.00	0.76	0.00	1.00	0.00	0.86	0.00			
	5	0.64	0.03	0.55	0.05	0.78	0.03	0.72	0.02	0.75	0.02			
	6	0.80	0.01	0.65	0.05	0.82	0.03	0.93	0.06	0.87	0.01			
	7	0.82	0.05	0.80	0.07	0.92	0.04	0.84	0.04	0.88	0.03	n.a.	n.a.	
	8	0.77	0.06	0.65	0.10	0.83	0.05	0.89	0.05	0.86	0.04			
**Depression [52]**	1	0.77	0.01	0.76	0.01	0.70	0.01	1.00	0.00	0.82	0.01	yes	<0.001	0.706 (0.586-0.812)
	2	0.77	0.02	0.76	0.03	0.69	0.02	1.00	0.00	0.82	0.02			
	3	0.68	0.02	0.67	0.03	0.62	0.02	1.00	0.00	0.76	0.01			
	4	0.52	0.01	0.50	0.01	0.52	0.01	1.00	0.00	0.69	0.01	yes	<0.001	
	5	0.52	0.01	0.50	0.01	0.52	0.01	1.00	0.00	0.69	0.01			
	6	0.59	0.04	0.57	0.04	0.56	0.03	1.00	0.00	0.72	0.02			
	7	0.87	0.07	0.87	0.08	0.81	0.09	0.97	0.03	0.89	0.06	n.a.	n.a.	
	8	0.61	0.08	0.59	0.08	0.57	0.05	0.98	0.00	0.72	0.04			
**Mania [9]**	1	0.78	0.01	0.88	0.01	0.29	0.01	1.00	0.00	0.45	0.02	yes	<0.001	0.363 (0.122-0.556)
	2	0.81	0.02	0.90	0.01	0.32	0.03	1.00	0.00	0.49	0.03			
	3	0.79	0.01	0.89	0.01	0.30	0.01	1.00	0.00	0.47	0.02			
	4	0.58	0.02	0.77	0.01	0.18	0.01	1.00	0.00	0.30	0.01			
	5	0.65	0.09	0.81	0.05	0.21	0.04	1.00	0.00	0.34	0.06			
	6	0.30	0.03	0.62	0.02	0.11	0.00	1.00	0.00	0.21	0.01	yes	<0.001	
	7	0.88	0.03	0.94	0.02	0.44	0.06	1.00	0.00	0.61	0.06	n.a.	n.a.	
	8	0.79	0.06	0.80	0.09	0.27	0.07	0.81	0.16	0.41	0.09			
**Negative symptoms [6]**	1	0.36	0.01	0.66	0.01	0.09	0.00	1.00	0.00	0.16	0.00	yes	0.016	0.32 (0.066-0.542)
	2	0.39	0.02	0.68	0.01	0.09	0.00	1.00	0.00	0.16	0.01			
	3	0.16	0.04	0.55	0.02	0.07	0.00	1.00	0.00	0.12	0.00			
	4	0.07	0.01	0.50	0.01	0.06	0.00	1.00	0.00	0.11	0.00	yes	<0.001	

	5	0.80	0.03	0.66	0.02	0.15	0.02	0.50	0.00	0.23	0.03			
	6	0.41	0.04	0.64	0.11	0.08	0.02	0.89	0.24	0.15	0.03			
	7	0.46	0.03	0.71	0.02	0.10	0.00	1.00	0.00	0.18	0.01	n.a.	n.a.	
	8	0.34	0.03	0.55	0.21	0.07	0.03	0.78	0.48	0.12	0.06			
**Positive symptoms [47]**	1	0.82	0.02	0.82	0.02	0.79	0.04	0.84	0.03	0.81	0.02	no	0.333	0.725 (0.614-0.826)
	2	0.85	0.04	0.85	0.04	0.79	0.05	0.91	0.00	0.85	0.03			
	3	0.85	0.00	0.85	0.00	0.86	0.00	0.81	0.00	0.84	0.00			
	4	0.78	0.04	0.78	0.04	0.71	0.04	0.89	0.00	0.79	0.03			
	5	0.69	0.03	0.70	0.03	0.64	0.02	0.77	0.06	0.70	0.04			
	6	0.61	0.08	0.63	0.08	0.56	0.05	0.83	0.14	0.67	0.08			
	7	0.85	0.09	0.85	0.09	0.84	0.11	0.84	0.08	0.84	0.09	n.a.	n.a.	
	8	0.62	0.11	0.61	0.10	0.63	0.19	0.47	0.09	0.54	0.10	yes	<0.001	
**Self-endangerment [84]**	1	0.87	0.01	0.70	0.03	0.90	0.01	0.94	0.03	0.92	0.01	no	0.281	0.175 (0.015-0.329)
	2	0.82	0.00	0.74	0.00	0.92	0.00	0.86	0.00	0.89	0.00			
	3	0.84	0.03	0.59	0.09	0.87	0.03	0.95	0.00	0.91	0.01			
	4	0.78	0.03	0.69	0.02	0.91	0.00	0.82	0.03	0.86	0.02			
	5	0.75	0.05	0.74	0.06	0.94	0.02	0.75	0.05	0.83	0.04			
	6	0.79	0.02	0.66	0.10	0.90	0.04	0.85	0.05	0.87	0.02			
	7	0.82	0.08	0.74	0.10	0.92	0.03	0.86	0.07	0.89	0.05	n.a.	n.a.	
	8	0.78	0.10	0.54	0.17	0.85	0.05	0.90	0.07	0.87	0.06	yes	<0.001	
**Self-harm [14]**	1	0.54	0.01	0.73	0.01	0.23	0.01	1.00	0.00	0.38	0.01	yes	<0.001	0.527 (0.335-0.688)
	2	0.54	0.14	0.73	0.08	0.24	0.05	1.00	0.00	0.38	0.07			
	3	0.65	0.05	0.80	0.03	0.29	0.03	1.00	0.00	0.44	0.04			
	4	0.54	0.17	0.73	0.10	0.24	0.07	1.00	0.00	0.38	0.09			
	5	0.37	0.11	0.59	0.07	0.17	0.02	0.90	0.20	0.29	0.04			
	6	0.24	0.32	0.56	0.19	0.16	0.06	1.00	0.00	0.27	0.09	yes	<0.001	
	7	0.48	0.02	0.67	0.07	0.20	0.02	0.93	0.18	0.33	0.04	yes	<0.001	
	8	0.54	0.17	0.72	0.11	0.23	0.07	0.98	0.10	0.38	0.09			

**Sleep [49]**	1	0.53	0.04	0.54	0.04	0.51	0.02	0.82	0.03	0.63	0.03	yes	<0.001	0.707 (0.609-0.794)
	2	0.67	0.09	0.68	0.09	0.63	0.07	0.80	0.11	0.71	0.09			
	3	0.52	0.10	0.52	0.10	0.50	0.07	0.79	0.08	0.62	0.07			
	4	0.55	0.41	0.55	0.40	0.58	0.38	0.58	0.08	0.57	0.18			
	5	0.54	0.02	0.54	0.04	0.53	0.02	0.62	0.65	0.55	0.24			
	6	0.52	0.01	0.52	0.02	0.51	0.01	0.58	0.47	0.53	0.19	yes	<0.001	
	7	0.77	0.07	0.77	0.07	0.70	0.06	0.93	0.08	0.80	0.07	n.a.	n.a.	
	8	0.61	0.09	0.62	0.09	0.56	0.05	0.96	0.05	0.71	0.06			
**Suicidality [58]**	1	0.93	0.00	0.92	0.00	0.92	0.00	0.97	0.00	0.94	0.00	n.a.	n.a.	0.733 (0.629-0.828)
	2	0.91	0.04	0.90	0.05	0.90	0.08	0.95	0.04	0.92	0.03			
	3	0.92	0.01	0.91	0.01	0.89	0.00	0.99	0.02	0.94	0.01			
	4	0.75	0.11	0.73	0.12	0.74	0.09	0.89	0.07	0.81	0.08			
	5	0.78	0.06	0.77	0.05	0.79	0.02	0.85	0.10	0.82	0.06			
	6	0.69	0.01	0.63	0.03	0.65	0.02	0.98	0.07	0.78	0.01	yes	<0.001	
	7	0.90	0.07	0.90	0.07	0.91	0.05	0.92	0.07	0.91	0.06	no	0.252	
	8	0.82	0.05	0.80	0.06	0.80	0.05	0.93	0.05	0.86	0.04			

GPT-OSS-120B

Condition [total No./100]	Prompt	Accuracy	95% CI	BAcc	95% CI	Precision	95% CI	Recall	95% CI	F1	95% CI	significant ΔBAcc vs. best prompt	p-value	IRR Fleiss' κ (95% CI)
**Addiction [38]**	1	0.87	0.08	0.88	0.07	0.76	0.11	0.96	0.04	0.85	0.08	no	0.074	0.678 (0.577-0.774)
	2	0.87	0.02	0.89	0.03	0.75	0.03	0.99	0.04	0.85	0.03			
	3	0.87	0.01	0.89	0.02	0.76	0.02	0.96	0.04	0.85	0.02			
	4	0.87	0.03	0.90	0.03	0.75	0.03	0.99	0.04	0.86	0.03			
	5	0.84	0.04	0.85	0.05	0.75	0.02	0.88	0.10	0.81	0.05			
	6	0.84	0.04	0.85	0.05	0.75	0.05	0.87	0.07	0.80	0.05	yes	0.023	
	7	0.92	0.07	0.93	0.07	0.86	0.14	0.95	0.07	0.90	0.09			
	8	0.91	0.00	0.92	0.01	0.82	0.03	0.98	0.04	0.89	0.00			
**Aggression [25]**	1	0.90	0.01	0.89	0.04	0.76	0.05	0.87	0.11	0.81	0.04	no	0.313	0.733 (0.603-0.844)
	2	0.90	0.02	0.88	0.03	0.77	0.08	0.85	0.06	0.81	0.04	no	0.337	
	3	0.91	0.02	0.89	0.02	0.81	0.08	0.84	0.00	0.82	0.04			
	4	0.89	0.03	0.89	0.03	0.72	0.06	0.91	0.06	0.80	0.05			
	5	0.86	0.04	0.89	0.03	0.66	0.07	0.95	0.06	0.78	0.05			
	6	0.89	0.04	0.92	0.03	0.69	0.07	1.00	0.00	0.82	0.05			
	7	0.91	0.01	0.89	0.03	0.78	0.01	0.87	0.06	0.82	0.03			
	8	0.91	0.04	0.91	0.05	0.79	0.09	0.89	0.06	0.84	0.08			
**Anxiety [31]**	1	0.84	0.01	0.86	0.01	0.67	0.02	0.94	0.00	0.78	0.01	no	0.250	0.338 (0.239-0.438)
	2	0.84	0.04	0.87	0.04	0.67	0.05	0.95	0.05	0.78	0.05			
	3	0.80	0.03	0.84	0.02	0.62	0.04	0.92	0.05	0.74	0.02	yes	0.012	
	4	0.83	0.04	0.86	0.04	0.66	0.05	0.95	0.05	0.78	0.05			
	5	0.83	0.01	0.85	0.02	0.66	0.01	0.92	0.05	0.77	0.02			
	6	0.83	0.01	0.86	0.01	0.66	0.02	0.94	0.00	0.78	0.01			
	7	0.86	0.04	0.88	0.03	0.70	0.07	0.93	0.00	0.80	0.05			
	8	0.85	0.04	0.88	0.05	0.70	0.04	0.94	0.08	0.80	0.06			
**Cognition**	1	0.92	0.04	0.93	0.03	0.99	0.00	0.91	0.06	0.95	0.03	n.a.	n.a.	0.516 (0.352-

**[76]**	2	0.92	0.06	0.91	0.06	0.97	0.02	0.92	0.07	0.95	0.04			0.656)
	3	0.92	0.04	0.91	0.02	0.96	0.00	0.94	0.05	0.95	0.03			
	4	0.94	0.01	0.90	0.01	0.95	0.00	0.97	0.02	0.96	0.01			
	5	0.70	0.11	0.52	0.13	0.77	0.06	0.85	0.10	0.81	0.07	yes	<0.001	
	6	0.67	0.12	0.55	0.22	0.78	0.11	0.79	0.03	0.79	0.07			
	7	0.88	0.03	0.92	0.04	0.99	0.02	0.85	0.02	0.91	0.02			
	8	0.85	0.01	0.88	0.05	0.98	0.04	0.82	0.03	0.89	0.01			
**Depression [52]**	1	0.87	0.04	0.87	0.04	0.82	0.04	0.97	0.03	0.89	0.03	yes	0.030	0.706 (0.586-0.812)
	2	0.90	0.01	0.89	0.01	0.85	0.00	0.97	0.03	0.91	0.01			
	3	0.87	0.01	0.87	0.01	0.81	0.02	0.99	0.03	0.89	0.01			
	4	0.92	0.01	0.92	0.01	0.89	0.00	0.97	0.03	0.93	0.01			
	5	0.88	0.04	0.88	0.04	0.82	0.04	0.99	0.03	0.90	0.04			
	6	0.86	0.03	0.86	0.03	0.81	0.04	0.96	0.00	0.88	0.02	yes	0.005	
	7	0.90	0.03	0.89	0.03	0.85	0.02	0.97	0.03	0.91	0.03			
	8	0.89	0.05	0.89	0.05	0.84	0.08	0.97	0.03	0.90	0.04			
**Mania [9]**	1	0.93	0.00	0.73	0.07	0.65	0.06	0.48	0.16	0.55	0.08	no	0.329	0.363 (0.122-0.556)
	2	0.93	0.02	0.74	0.08	0.64	0.18	0.52	0.16	0.57	0.16			
	3	0.92	0.09	0.79	0.19	0.55	0.49	0.63	0.32	0.58	0.42			
	4	0.91	0.04	0.82	0.06	0.51	0.20	0.70	0.16	0.59	0.11			
	5	0.92	0.03	0.71	0.02	0.61	0.24	0.44	0.00	0.51	0.09	no	0.136	
	6	0.94	0.02	0.72	0.01	0.82	0.42	0.44	0.00	0.57	0.10			
	7	0.95	0.01	0.72	0.01	0.93	0.29	0.44	0.00	0.60	0.06			
	8	0.94	0.02	0.72	0.01	0.82	0.42	0.44	0.00	0.57	0.10			
**Negative symptoms [6]**	1	0.77	0.01	0.88	0.01	0.21	0.01	1.00	0.00	0.35	0.01	no	0.289	0.32 (0.066-0.542)
	2	0.83	0.05	0.91	0.03	0.26	0.06	1.00	0.00	0.42	0.07			
	3	0.42	0.09	0.69	0.05	0.09	0.01	1.00	0.00	0.17	0.02	yes	<0.001	
	4	0.75	0.05	0.87	0.03	0.19	0.03	1.00	0.00	0.32	0.04			
	5	0.74	0.03	0.86	0.02	0.19	0.02	1.00	0.00	0.32	0.02			

	6	0.73	0.04	0.86	0.02	0.18	0.02	1.00	0.00	0.31	0.03			
	7	0.90	0.04	0.92	0.12	0.37	0.12	0.94	0.24	0.53	0.16			
	8	0.90	0.10	0.92	0.16	0.37	0.24	0.94	0.24	0.53	0.29			
**Positive symptoms [47]**	1	0.92	0.03	0.92	0.03	0.93	0.06	0.89	0.00	0.91	0.03	no	0.289	0.725 (0.614-0.826)
	2	0.91	0.01	0.91	0.02	0.91	0.00	0.90	0.03	0.91	0.02			
	3	0.87	0.01	0.87	0.02	0.83	0.00	0.91	0.03	0.86	0.02	yes	0.013	
	4	0.91	0.01	0.91	0.01	0.90	0.03	0.90	0.03	0.90	0.02			
	5	0.93	0.07	0.93	0.07	0.93	0.06	0.91	0.09	0.92	0.08			
	6	0.91	0.00	0.91	0.00	0.91	0.02	0.90	0.03	0.90	0.00			
	7	0.93	0.02	0.92	0.01	0.95	0.03	0.89	0.00	0.92	0.02			
	8	0.90	0.02	0.90	0.01	0.93	0.05	0.86	0.03	0.89	0.01			
**Self-endangerment [84]**	1	0.85	0.04	0.74	0.02	0.92	0.01	0.91	0.05	0.91	0.03	no	0.398	0.175 (0.015-0.329)
	2	0.86	0.04	0.75	0.05	0.92	0.02	0.91	0.05	0.91	0.02			
	3	0.86	0.00	0.72	0.07	0.91	0.03	0.92	0.03	0.92	0.00			
	4	0.85	0.09	0.75	0.12	0.92	0.04	0.90	0.07	0.91	0.05			
	5	0.82	0.06	0.65	0.13	0.89	0.04	0.90	0.03	0.90	0.04	yes	0.010	
	6	0.82	0.11	0.67	0.19	0.90	0.06	0.89	0.07	0.89	0.07			
	7	0.84	0.06	0.75	0.08	0.93	0.03	0.88	0.05	0.90	0.04			
	8	0.84	0.03	0.75	0.05	0.92	0.02	0.89	0.02	0.90	0.02			
**Self-harm [14]**	1	0.56	0.05	0.74	0.03	0.24	0.02	1.00	0.00	0.39	0.03	no	0.109	0.527 (0.335-0.688)
	2	0.54	0.06	0.73	0.03	0.23	0.02	1.00	0.00	0.38	0.03			
	3	0.89	0.04	0.88	0.03	0.57	0.12	0.86	0.00	0.69	0.09			
	4	0.62	0.01	0.78	0.01	0.27	0.01	1.00	0.00	0.42	0.01			
	5	0.44	0.06	0.68	0.04	0.20	0.02	1.00	0.00	0.33	0.03	yes	0.005	
	6	0.44	0.06	0.68	0.04	0.20	0.02	1.00	0.00	0.33	0.03			
	7	0.62	0.05	0.77	0.07	0.27	0.04	0.98	0.10	0.42	0.06			
	8	0.60	0.03	0.77	0.02	0.26	0.02	1.00	0.00	0.41	0.02			
**Sleep**	1	0.83	0.03	0.83	0.03	0.84	0.03	0.80	0.03	0.82	0.03	no	0.313	0.707 (0.609-

**[49]**	2	0.84	0.02	0.84	0.02	0.84	0.04	0.84	0.00	0.84	0.02			0.794)
	3	0.82	0.04	0.82	0.04	0.83	0.08	0.80	0.03	0.82	0.03			
	4	0.83	0.01	0.83	0.01	0.86	0.03	0.78	0.00	0.81	0.01			
	5	0.80	0.05	0.80	0.05	0.77	0.05	0.83	0.06	0.80	0.05	yes	0.006	
	6	0.80	0.02	0.80	0.02	0.77	0.04	0.84	0.08	0.81	0.03			
	7	0.84	0.04	0.84	0.04	0.83	0.03	0.84	0.08	0.84	0.05			
	8	0.83	0.02	0.83	0.02	0.83	0.06	0.82	0.03	0.82	0.01			
**Suicidality [58]**	1	0.90	0.04	0.89	0.04	0.89	0.02	0.94	0.07	0.92	0.04	no	0.074	0.733 (0.629-0.828)
	2	0.89	0.01	0.89	0.02	0.89	0.02	0.93	0.00	0.91	0.01			
	3	0.92	0.02	0.91	0.03	0.88	0.05	0.99	0.02	0.94	0.02			
	4	0.94	0.05	0.93	0.05	0.93	0.03	0.97	0.07	0.95	0.04			
	5	0.88	0.06	0.87	0.07	0.85	0.07	0.98	0.02	0.91	0.04			
	6	0.88	0.02	0.86	0.03	0.85	0.03	0.97	0.04	0.90	0.02	yes	0.005	
	7	0.91	0.04	0.91	0.04	0.92	0.03	0.91	0.04	0.92	0.03			
	8	0.82	0.05	0.80	0.06	0.80	0.05	0.93	0.05	0.86	0.04			

Model name in the headline. Prompt 1: baseline, 2: role+baseline, 3: role+definition+baseline, 4: role+definition+examples+baseline, 5: scrambled (baseline), 6: extra scrambled (role+baseline), 7: Chain-of-Thought (CoT), 8: Reflection-of-Thought. BAcc - Balanced accuracy. IRR - Inter-rater reliability. Best (green) and worst (red) BAcc highlighted for each domain. Means, 95%-CIs and p-values derived from 2,000 bootstrapping iterations. P-values were Benjamini-Hochberg-adjusted across all domains and metrics (false-discovery-rate ≤ 0.05). Significance of BAcc-differences between the best and worst prompt, between the best and baseline prompt, and between the best and CoT prompt are given in the respective row, where applicable. Fleiss κ ranges: 0 - 0.20 → slight, 0.21 - 0.40 → fair, 0.41 - 0.60 → moderate, 0.61 - 0.80 → substantial, 0.81 - 1 → almost perfect.

Supplementary Table S3: Performance metrics, significance levels and feature weights for diagnostic group classifiers

Llama-3.1-70B																								
**classifier**	Dementia (F0)				SUD (F1)				Psychotic Disorders (F2)				Affective Disorders (F3)				Anxiety Disorders (F4)				Personality Disorders (F5)			
**mean BACC**	82.25		75.07		78.86		80.23		74.97		64.31		69.67		70.85		68.05		65.34		83.11		75.84	
**95% CI**	1.81		3.61		1.27		1.76		2.35		2.80		2.09		0.98		4.76		3.84		3.66		4.02	
**p value**	0.004				0.046				<0.001				0.247				0.340				<0.001			
	**Feature weights (mean, S.E.)**																							
**Addiction**	-0.54	0.01	-0.63	0.06	1.00	0.00	1.00	0.00	0.00	0.00	-0.03	0.09	-0.12	0.09	-0.61	0.04	-0.48	0.05	-0.55	0.06	0.00	0.00	-0.03	0.09
**Aggression**	0.31	0.01	0.15	0.09	0.00	0.00	0.00	0.00	0.00	0.00	0.00	0.01	-0.16	0.11	-0.24	0.05	-0.31	0.08	-0.15	0.16	0.00	0.00	0.00	0.00
**Anxiety**	-0.28	0.01	-0.03	0.03	0.00	0.00	0.00	0.00	0.00	0.00	0.01	0.02	0.06	0.06	-0.01	0.02	0.17	0.03	0.00	0.00	0.00	0.00	0.44	0.13
**Cognition**	0.56	0.01	0.62	0.04	0.00	0.00	0.00	0.00	0.00	0.00	-0.01	0.03	-0.06	0.06	-0.30	0.07	-0.19	0.06	-0.06	0.13	0.00	0.00	-0.49	0.06
**Depression**	-0.25	0.01	-0.06	0.03	0.00	0.00	0.00	0.00	0.00	0.00	0.00	0.01	0.51	0.15	-0.04	0.03	0.12	0.05	0.42	0.12	0.00	0.00	0.45	0.13
**Mania**	-0.01	0.01	0.06	0.03	0.00	0.00	0.00	0.00	0.00	0.00	0.03	0.08	0.16	0.11	-0.26	0.03	-0.02	0.10	-0.06	0.13	-0.54	0.10	0.00	0.00
**Negative symptoms**	-0.04	0.01	-0.05	0.03	0.00	0.00	0.00	0.00	0.00	0.00	-0.01	0.02	-0.01	0.02	0.00	0.00	-0.36	0.05	0.33	0.18	0.00	0.00	-0.01	0.03
**Positive symptoms**	0.02	0.01	-0.06	0.03	0.00	0.00	0.00	0.00	1.00	0.00	0.99	0.03	-0.73	0.02	-0.58	0.04	-0.62	0.04	-0.50	0.07	0.00	0.00	0.00	0.00
**Self-endangerment**	-0.02	0.01	0.00	0.00	0.00	0.00	0.00	0.00	0.00	0.00	0.01	0.03	-0.01	0.02	0.00	0.00	0.07	0.03	0.00	0.00	0.01	0.04	0.00	0.00
**Self-harm**	-0.26	0.01	-0.34	0.20	0.00	0.00	0.00	0.00	0.00	0.00	0.00	0.01	-0.08	0.06	0.01	0.02	0.08	0.06	0.02	0.05	0.60	0.06	0.55	0.11
**Sleep**	-0.04	0.02	-0.04	0.03	0.00	0.00	0.00	0.00	0.00	0.00	-0.01	0.04	0.03	0.05	0.01	0.02	0.10	0.07	0.03	0.06	0.00	0.00	0.00	0.00
**Suicidality**	-0.28	0.01	-0.03	0.03	0.00	0.00	0.00	0.00	0.00	0.00	-0.01	0.04	0.17	0.12	0.25	0.04	0.11	0.04	0.03	0.05	0.58	0.01	0.02	0.04

GPT-OSS-120B																								
**classifier**	Dementia (F0)				SUD (F1)				Psychotic Disorders (F2)				Affective Disorders (F3)				Anxiety Disorders (F4)				Personality Disorders (F5)			
**mean BACC**	81.90		79.72		79.69		78.68		73.09		73.05		75.86		78.02		70.87		65.52		76.68		73.23	
**95% CI**	2.18		3.75		0.70		0.96		2.38		2.03		1.46		1.01		4.55		3.35		3.75		3.38	
**p value**	0.307				0.109				0.950				0.002				0.009				0.032			
	**Feature weights (mean, S.E.)**																							
**Addiction**	-0,35	0,03	-0,54	0,03	1,00	0,00	0,74	0,09	0,00	0,00	0,00	0,00	0,00	0,00	-0,30	0,04	-0,55	0,03	-0,46	0,06	-0,02	0,05	0,02	0,04
**Aggression**	0,19	0,04	0,38	0,03	0,00	0,00	0,00	0,00	0,00	0,00	0,00	0,00	-0,58	0,00	-0,31	0,02	-0,12	0,08	-0,22	0,10	-0,08	0,06	0,02	0,05
**Anxiety**	-0,14	0,03	-0,18	0,04	0,00	0,00	0,00	0,00	0,00	0,00	0,00	0,00	0,00	0,00	0,02	0,03	0,35	0,04	0,35	0,07	0,12	0,04	0,17	0,06
**Cognition**	0,63	0,06	0,56	0,02	0,00	0,00	0,00	0,00	0,00	0,00	0,00	0,00	0,00	0,00	-0,01	0,02	-0,10	0,08	0,09	0,09	-0,30	0,06	-0,17	0,06
**Depression**	-0,20	0,04	-0,21	0,04	0,00	0,00	0,00	0,00	0,00	0,00	0,00	0,00	0,58	0,00	0,33	0,02	-0,01	0,01	0,27	0,08	-0,11	0,04	0,23	0,07
**Mania**	-0,14	0,05	-0,15	0,06	-0,02	0,04	-0,65	0,18	0,00	0,00	0,00	0,00	0,58	0,00	0,65	0,04	-0,36	0,10	-0,35	0,07	-0,02	0,09	-0,04	0,05
**Negative symptoms**	-0,02	0,03	-0,16	0,02	0,00	0,00	0,00	0,00	0,00	0,00	0,00	0,00	0,00	0,00	0,31	0,01	-0,24	0,06	-0,26	0,08	-0,21	0,08	-0,40	0,09
**Positive symptoms**	0,02	0,02	0,06	0,04	0,00	0,00	0,00	0,00	1,00	0,00	1,00	0,00	0,00	0,00	-0,31	0,02	-0,35	0,04	-0,46	0,07	-0,17	0,05	-0,08	0,07
**Self-endangerment**	0,01	0,02	0,07	0,09	0,00	0,00	0,00	0,00	0,00	0,00	0,00	0,00	0,00	0,00	0,00	0,00	0,00	0,01	0,04	0,07	0,41	0,04	0,26	0,06
**Self-harm**	-0,55	0,15	-0,24	0,04	0,00	0,00	0,00	0,00	0,00	0,00	0,00	0,00	0,00	0,00	0,00	0,00	0,20	0,05	-0,09	0,09	0,58	0,07	0,67	0,06
**Sleep**	-0,06	0,06	-0,11	0,09	0,00	0,00	0,00	0,00	0,00	0,00	0,00	0,00	0,00	0,00	0,29	0,05	0,22	0,04	0,09	0,10	0,17	0,05	0,04	0,05
**Suicidality**	-0,14	0,04	-0,14	0,05	0,00	0,00	0,00	0,00	0,00	0,00	0,00	0,00	0,00	0,00	0,02	0,03	0,35	0,04	0,17	0,12	0,48	0,03	0,41	0,05

Model name in the headline. Performance metrics across all diagnostic group-vs-other classifiers with 95% CIs. Feature weights given with standard error. Green columns are results from “best” prompts and red from “worst”. P-values for two-sided t-test comparison between “best” and “worst” models with α = 0.05. SUD - Substance Use Disorders.

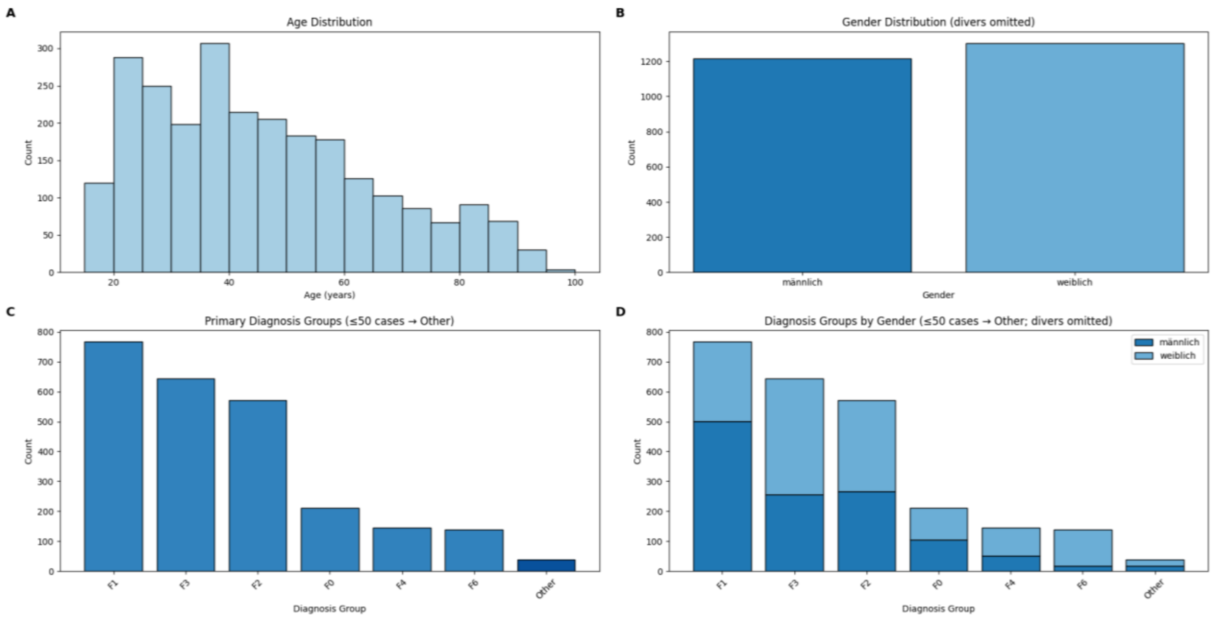

Supplementary Figure S1: Distribution of age, gender and primary diagnoses in 2.520 case samples from 1,692 patients. (A) Age distribution, mean age 45.2 ± 0.76 years, mean female age 45.95 ± 1.11 years, mean male age 44.43 ± 1.02 years. (B) Gender distribution, female: 1303 cases, male: 1215 cases, non-binary: 2 cases (not shown). (C) Primary diagnostic ICD-10 group, by frequency: F1 (substance use, any kind) 767 cases, F3 (affective disorders) 645 cases, F2 (Schizophrenia, schizotypal and delusional disorders) 572 cases, F0 (organic mental disorders, including dementia) 211 cases, F4 (anxiety disorders) 146 cases, F6 (disorders of adult personality and behavior) 141 cases, 38 other cases. (D) gender balance by main diagnostic groups (ratio = male/female, rounded): $F0_{ratio}$ = 1:1 (0.99), $F1_{ratio}$ = 2:1 (1.89), $F2_{ratio}$ = 1:1 (0.88), $F3_{ratio}$ = 2:3 (0.66), $F4_{ratio}$ = 1:2 (0.54), $F6_{ratio}$ = 1:7 (0.14).

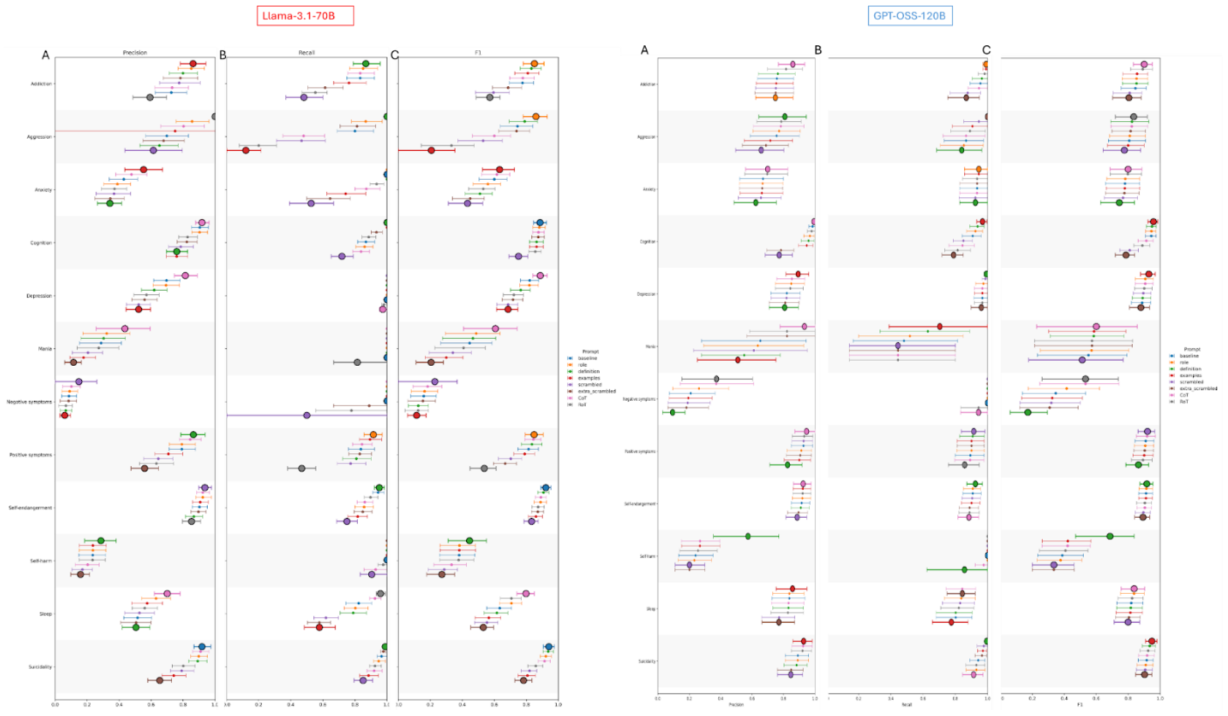

Supplementary Figure S2: Performance metrics for both Llama and GPT models across all domains showing results for all prompts, highlighting best and worst prompting strategies (bold), error bars marking 95%-CIs from 2,000-fold bootstrapping across three independent experimental runs. (A) Precision, (B) Recall, (C) F1-score.

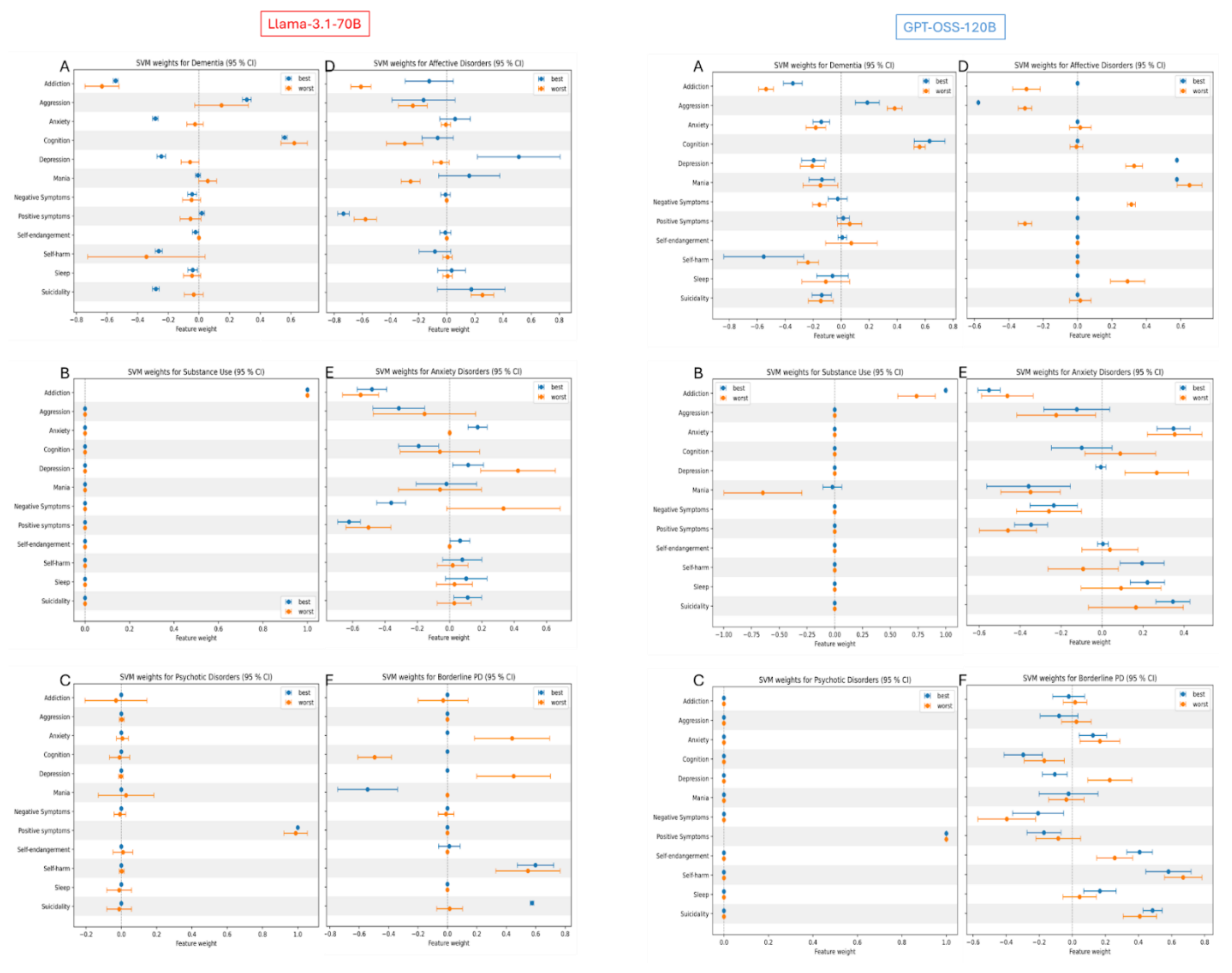

Supplementary Figure S3: All feature weights for the machine learning classifiers, respectively for Llama and GPT models. Shown for “best” (blue) vs. “worst” LLM-extraction strategies. Psychotic disorders and affective disorders as shown in figure 2B&C. Larger feature weight total values indicate stronger importance in SVM classification. Bars with whiskers indicate 95%-CIs. Corresponding BAcc as reference for Llama (A) best: 82.25%, worst: 75.07%, (B) best: 78.86%, worst: 80.23%, (C) best: 74.97%, worst: 64.31%, (D) best: 69.67% worst: 70.85%, (E) best: 68.05% worst: 65.34%, (F) best: 83.11% worst: 75.84%. For GPT (A) best: 81.90%, worst: 79.72%, (B) best: 79.69%, worst: 78.68%, (C) best: 73.09%, worst: 73.05%, (D) best: 75.86% worst: 78.02%, (E) best: 70.86% worst: 65.52%, (F) best: 76.68% worst: 73.23%.
